# The combination of morphogenic regulators BABY BOOM and GRF‐GIF improves maize transformation efficiency and promotes leaf regeneration

**DOI:** 10.1111/nph.71508

**Published:** 2026-08-17

**Authors:** Zongliang Chen, Jiaqi Zhou, Mary Galli, Sessen Daniel Iohannes, Theresa Clark, Juan M. Debernardi, Jorge Dubcovsky, David Jackson, Andrea Gallavotti

**Affiliations:** ^1^ Waksman Institute of Microbiology Rutgers University Piscataway NJ 08854‐8020 USA; ^2^ Cold Spring Harbor Laboratory Cold Spring Harbor NY 11724 USA; ^3^ Department of Plant Sciences University of California Davis Davis CA 95616 USA; ^4^ Howard Hughes Medical Institute Chevy Chase MD 20815 USA; ^5^ Department of Plant Biology Rutgers University New Brunswick NJ 08901 USA

**Keywords:** BABY BOOM, GGB and GB transformation systems, GRF‐GIF, leaf regeneration, maize, WUSCHEL

## Abstract

- Transformation is an indispensable tool for plant genetics and functional genomics. Although stable transformation in maize is no longer a major obstacle, there remains a need for accessible and efficient methods for academic laboratories.
- Here, we present the GGB system, a rapid and efficient approach optimized for immature embryo transformation in B104 and other maize lines. This system combines two distinct morphogenetic regulators, the wheat GRF4‐GIF1 chimera and the maize BABY BOOM (BBM) transcription factor (hence the name “GGB”) with a modified QuickCorn protocol, enabling regeneration of transformed maize plantlets in *c*. 2 months with an efficiency 7‐fold higher than when compared to either morphogenic factor used in isolation.
- Expression of both regulators did not significantly affect development, eliminating the need to excise them after regeneration. However, transmission of the transgenic GGB construct through pollen was significantly reduced, potentially aiding transgenic line containment. We show that the GGB system is adaptable for CRISPR‐Cas9 editing and reporter line generation. Furthermore, stable GGB transformants exhibited high leaf regeneration capacity via somatic embryogenesis.
- RNA‐seq time‐course profiling of GGB leaf cultures identified additional factors that could promote regeneration and led to the discovery of asparagine and trehalose as additional media components that significantly enhanced leaf regeneration.

## Graphical abstract

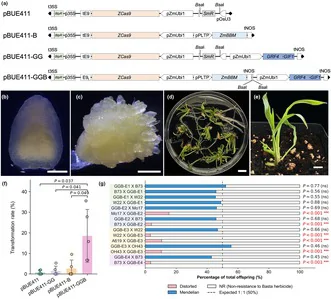

## Introduction

The promise of genome editing to rapidly advance crop improvement relies for most species on the ability to generate transformed plants. However, lengthy and inefficient methods for transformation and regeneration of recalcitrant species as well as the genotype dependency of the transformation process hinder the widespread adoption of crop transformation technologies. For most species, simple transformation systems, such as floral dipping and *in planta* injection followed by *de novo* organ formation, are not a viable strategy. Tissue culture‐based transformation systems, which rely on callus induction and optimized phytohormone ratios, remain the only approaches for regenerating transgenic plants in many crop species. These systems require extensive empirical testing of media components, particularly phytohormone types and their concentrations, and are inherently time‐consuming. Furthermore, they often carry unintended consequences (e.g. somaclonal variation; Phillips *et al*., 1994; Neelakandan & Wang, 2012) and require specialized training.

Plant regeneration is a common feature across diverse plant lineages and a hallmark of development, yet cereals remain notably recalcitrant, with major food crops such as rice and maize exhibiting limited regenerative ability from somatic tissues (Ikeuchi *et al*., 2019). The use of various morphogenic factors, genes that are involved in embryogenesis or meristem development, can trigger reprogramming of a subset of somatic cells to eventually produce transformed plants (Gordon‐Kamm *et al*., 2019; Kausch *et al*., 2019; Maren *et al*., 2022). Inducing regeneration‐competent or embryogenic cells is a critical step for plant transformation, and it has long been known that phytohormones, auxin and cytokinin, as well as wounding are the original triggering signals for plant regeneration (Chen *et al*., 2022). Key morphogenic regulators have been successfully manipulated to induce regeneration in crop plants. The most significant advancement was the introduction of the WUSCHEL2‐BABY BOOM (WUS‐BBM) system in maize and other crops by Corteva (Lowe *et al*., 2016, 2018). This system relies on the ectopic expression of two developmental regulators involved in meristem activity, the maize *ZmWUS2* and *ZmBBM/EREB53* genes. *ZmWUS2* is a maize co‐ortholog of the Arabidopsis *WUS* gene (maize has another co‐ortholog called *ZmWUS1*; Nardmann & Werr, 2006). The Arabidopsis *WUS* gene is required for shoot apical meristem establishment during embryogenesis, for axillary meristem initiation postembryogenesis, and for meristem maintenance (Mayer *et al*., 1998; Lenhard *et al*., 2002; Wang *et al*., 2017). *BBM* encodes an AP2/EREB transcription factor. Overexpression of *BBM* homologs in different plant species has been used to boost somatic embryogenesis in vegetative tissue (Boutilier *et al*., 2002; Deng *et al*., 2009; Heidmann *et al*., 2011). However, due to pleiotropic effects, removal of both morphogenic regulators from transgenic plants is required in this system (Lowe *et al*., 2018; Wang *et al*., 2020b). Alternative strategies, including tissue‐specific promoters or nonintegrating expression, have been pursued to obviate this issue (Lowe *et al*., 2018; Hoerster *et al*., 2020; Che *et al*., 2022) and these have been implemented for the efficient transformation of the recalcitrant B73 inbred as well (Kang *et al*., 2023). Recently, when combined with *Agrobacterium* strains carrying a helper plasmid (an improved ternary vector carrying additional virulence genes), another member of *WUSCHEL* family, *TaWOX5*, was found to increase transformation efficiency of the maize inbred lines B73 and A188, generating transgenic plants without obvious developmental defects (Wang *et al*., 2022a). The use of *WOX2a* in maize was also shown to enable transformation of recalcitrant genotypes (McFarland *et al*., 2023).

The constitutive expression of the wheat *GROWTH REGULATING FACTOR4 – GRF‐INTERACTING FACTOR1* (*GRF4‐GIF1*) chimeric gene has been shown to enhance transformation efficiency in wheat and other monocot species (Debernardi *et al*., 2020; Vandeputte *et al*., 2024). Additionally, two individual maize GRFs (*ZmGRF5‐LIKE1* and *ZmGRF5‐LIKE2*) have been reported to boost transformation efficiency in the maize inbred A188 without pleiotropic effects in transgenic plants (Kong *et al*., 2020). As important developmental regulators, GRFs together with GIFs hold potential for improving transformation efficiency of different plant species (Debernardi *et al*., 2014, 2020).

Despite the recent breakthroughs, challenges still exist in developing fast, efficient and reliable transformation systems that can be quickly adopted by the public sector for basic and applied research, in particular for monocot species. In this study, we combined the morphogenic regulators ZmBBM and TaGRF4‐GIF1 in a single binary vector carrying Cas9 to explore the potential of this combination to enhance transformation efficiency and CRISPR‐mediated editing in maize inbred line B104, commonly used by transformation facilities. In combination with a modified QuickCorn protocol (Masters *et al*., 2020), we found that this system significantly enhanced the efficiency of transformation in B104, allowing the generation of transgenic plants within a two‐month time frame. Similar results were obtained with two additional transformable backgrounds, Hi‐II and LH244 (Tan *et al*., 2026). We named this transformation system GGB, for GRF‐GIF‐BBM, and it proved to be a rapid and highly efficient system that can be adopted by academic laboratories with little transformation experience. Stable transformed plants carrying BBM and TaGRF4‐GIF1 enabled plant regeneration from normally recalcitrant leaf tissues. To further investigate mechanisms underlying leaf regeneration in the GGB system, we performed time‐course RNA‐seq analysis. By comparing the regeneratable GGB line with the wild‐type (WT) B104, we identified potential positive regulators of regeneration. Among these, asparagine and trehalose supplements to the regeneration medium were found to enhance leaf‐based regeneration. This study adds to a growing list of tools available for functional genomic studies in maize (Lowe *et al*., 2016, 2018; Mookkan *et al*., 2017; Gordon‐Kamm *et al*., 2019; Hoerster *et al*., 2020; Masters *et al*., 2020; Kang *et al*., 2022) and provides a system that can be easily adopted for maize transformation by academic laboratories.

## Materials and Methods

### Plant materials

The maize (*Zea mays* L.) inbred B104, LH244, B73, A619 and W22 inbred lines and the Hi‐II line were grown in standard glasshouse conditions during winter and spring (2021–2025) and in summer nursery fields (2021–2025) located at the Waksman Institute of Microbiology in Piscataway, NJ, United States.

### Maize GGB vector construction

We performed all PCR cloning with Phusion High‐Fidelity DNA Polymerase (New England BioLabs, Ipswich, MA, USA). Total RNA was extracted from 3 to 5 mm ear primordia of the inbred line B73 using RNeasy Mini Kit (Qiagen) with on‐column DNase I (Qiagen) treatment and used for complementary DNA (cDNA) synthesis with a ProtoScript® II First Strand cDNA Synthesis Kit (New England BioLabs). To clone the coding region of maize *BABY BOOM* (*BBM*, GRMZM2G141638; Lowe *et al*., 2018), we performed PCR using cDNA generated from ear primordia, and the PCR product was cloned into the pENTR223 entry vector by SfiI digestion followed by sticky‐end ligation performed by the T4 DNA ligase (New England BioLabs). To clone the 1098 bp promoter region and 5’‐UTR of the PHOSPHOLIPID TRANSFERASE PROTEIN gene (*PLTP*, GRMZM2G101958; Lowe *et al*., 2018), we performed PCR using genomic DNA extracted from leaves of B73, and the PCR product was cloned into pTF101 vector by HindIII and BamHI digestion followed by sticky‐end ligation performed by the T4 DNA ligase. The coding region of maize *BBM* was then cloned into pTF101 vector containing the *PLTP* promoter sequence by LR Clonase II Enzyme Mix (Thermo Fisher Scientific, USA).

**Fig. 1:**
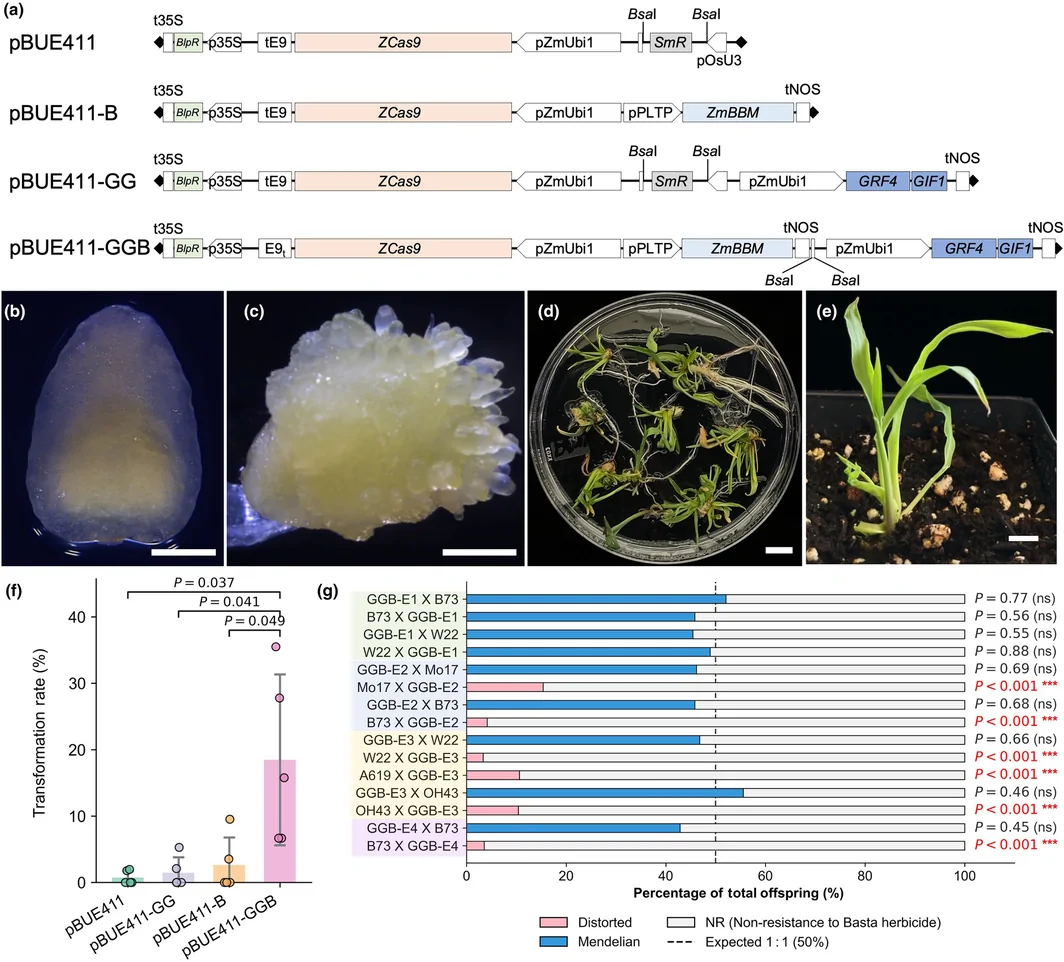
Maize regeneration via somatic embryogenesis by ectopic expression of morphogenic factors TaGRF4‐TaGIF1 and ZmBBM. (a) Diagrams of four binary vectors used for stable maize transformation. (b) Immature embryo of maize inbred line B104 used as explants. (c) Somatic embryos emerged on the surface of embryo scutellum side at 6 d after Agrobacterium infection (DAI). (d) Whole plantlet regeneration on rooting medium at 6 wk after infection. (e) Regenerated plantlet was acclimated to soil in growth chamber. (f) Transformation rate of constructs pBUE411, pBUE411‐TaGRF4‐GIF1, pBUE411‐BBM and pBUE411‐TaGRF4‐GIF1‐BBM using the inbred line B104. The average transformation efficiency for pBUE411 is 0.9% (fresh embryo number = 378, biological repeats = 4), pBUE411‐G is 1.8% (fresh embryo number = 234, biological repeats = 4), pBUE411‐B is 2.6% (fresh embryo number = 321, biological repeats = 5) and pBUE411‐GGB_B is 18.5% (fresh embryo number = 316, biological repeats = 5). pBUE411‐G, pBUE411‐TaGRF4‐GIF1; pBUE411‐B, pBUE411‐BBM, pBUE411‐GGB_B and pBUE411‐TaGRF4‐GIF1‐BBM transformed into B104 inbred lines. Each dot represents an independent experiment; error bars, SD; P, independent two‐sample *t*‐tests. (g) Skewed pollen transmission in GGB‐E2 to GGB‐E4 in reciprocal crosses with different inbred lines (B73, W22, Mo17, A619 and OH43). Bars, 0.5 mm in (b) and (c), 1 mm in (d) and (e). *** indicates statistically significant difference; ns indicates nonsignificance; P, chi‐squared test. p35S, Cauliflower Mosaic Virus 35S promoter; pOsU3, *Oryza sativa U3 small nuclear RNA* promoter; pPLTP, *Zea mays Phospholipid Transferase Protein* gene promoter; pZmUbi1, *Zea mays UBIQUITIN1* promoter; t35S, Cauliflower Mosaic Virus 35S terminator; tE9, rbcS‐E9 terminator; tNOS, Nopaline Octopine Synthase terminator.

To generate the GGB construct containing BBM and the GRF4‐GIF1 chimeric protein combined with Cas9 construct, we modified the available pBUE411 vector (Xing *et al*., 2014). In the first step, we performed PCR to clone *ZmUBIQUITIN*_pro_::*GRF4*‐*GIF1* using primers JD633‐F1/R1 and JD633 plasmid as templates (Debernardi *et al*., 2020). The resulting PCR product was cloned into pBUE411 vector at the PmeI cut site by Gibson Assembly using NEBuilder HiFi DNA Assembly Cloning Kit (New England BioLabs), and this produced the pBUE411_GRF‐GIF construct (Fig. 1a). Subsequently, the guide RNA cassette of pBUE411 was replaced with a synthetic fragment carrying a multiple cloning site, and then the PLTP_pro_::BBM‐NOS_term_ was amplified using the pTF101‐ PLTP_pro_::BBM‐NOS_term_ plasmid as template and cloned into the multiple cloning site. The resulting pBUE411_GRF‐GIF‐BBM construct contains ZmUbi_pro_::GRF4‐GIF1‐NOS_term_, PLTP_pro_::BBM‐NOS_term_ and ZmUbi_pro_::Cas9‐E9_term_ (Fig. 1a). To generate the pBUE411_BBM vector construct (pBUE411 PLTP_pro_::BBM‐NOS_term_), the pBUE411_GRF‐GIF‐BBM construct was cut by AflII enzyme on double AflII cut sites to remove *ZmUBIQUITIN*_pro_::*GRF4*‐*GIF1*, followed by self‐ligation using T4 DNA ligase (New England BioLabs; Fig. 1a). The pBUE411_GGB_M and pBUE411_GGB_N constructs were created by removing the Cas9 cassette using AscI and NruI sites, and the fluorescent reporter construct was inserted in those sites.

To incorporate the gRNAs into the pBUE411‐GGB construct we first generated two entry vectors that carry OsU3_pro_‐*Bsa*I_SmR_*Bsa*I‐scaffold‐OsU3_term_ and OsU6p2_pro_‐*Bsa*I_SmR_*Bsa*I‐scaffold‐OsU3_term_, respectively (Supporting Information Fig. S1). Individual gRNA fragments were then ligated into the double *BsaI* sites by T4 DNA ligase following *BsaI* digestion to generate individual gRNA cassettes. Single or multiple individual gRNA cassettes were PCR amplified using primer combinations in the primer list (Table S1) and assembled into double *BsaI* sites of pBUE411‐GGB construct (Fig. 1a) using Golden Gate Assembly method. For Golden Gate Assembly reactions (15 μl per reaction), equal molar concentrations of PCR products of gRNA cassettes and the pBUE411‐GGB plasmid were mixed in a single 200 μl Eppendorf tube. A total of 0.5 μl *BsaI* enzyme (New England BioLabs) and 1 μl T4 DNA ligase (New England BioLabs) were then added to the tube, and finally, MilliQ water was added to bring the final volume to 15 μl. Golden Gate Assembly reactions were setup in a PCR machine with 25–30 cycles of 37°C for 4 min and 16°C for 10 min. After the assembly reaction, the final plasmids were kept at 50°C for extra 5 min to linearize unexpected products carrying *BsaI* cut sites followed by 80°C for 10 min. One or two microliters of final reaction products were used for transformation of chemically competent *E. coli* cells.

Golden Gate Assembly, together with restriction enzyme digestion followed by T4 ligation, was used to generate the GB constructs, pGGB‐ZmUBI_pro_::mNG‐NLS and pGGB‐OCL4_pro_::YFPN7. The overall construct generation was carried out using the same reaction setup described above. All components were amplified with Phusion DNA polymerase, and the fragments were ordered and joined using Golden Gate Assembly. The assembled products were then subjected to restriction digestion and T4 ligation to obtain the final constructs.

### Maize transformation protocol

Maize transformation of immature embryos followed previously published protocols (Lowe *et al*., 2018; Masters *et al*., 2020) with a few modifications. For harvesting immature embryos, we grew Hi‐II, B104, LH244, B73, A619 and W22 plants in standard glasshouse or in field conditions. We harvested fresh ears (10–14 d after pollination) and removed all husk leaves and silks and then surface‐sterilized them for 20 min in 50% commercial bleach (1.65% sodium hypochlorite) with 0.1% of Tween‐20. After surface sterilization, we washed the ears three times with sterilized water and dissected the immature embryos in laminar flow cabinets (embryo sizes, 1.5–2.0 mm) using a sterilized spatula. The fresh embryos were collected in a 2‐ml microcentrifuge tube filled with infection medium (700A) and subsequently washed for three times with the same infection medium. For *Agrobacterium tumefaciens* inoculations, the EHA105 strain was used for the transformation of pBUE411, pBUE411‐GG, pBUE411‐B, pBUE41‐GGB, pGGB‐mNG‐NLS, pGGB‐pOCL4::YFPN7 and pGGB‐SAUR15/79‐gRNA vectors, while the LBA4404 strain carrying the pVS1‐VIR2 helper plasmid was used with the pGB vector. *Agrobacterium* strains stored as a glycerol stocks at −70°C were grown on a Yeast Extract Peptone (YEP) agar plate (peptone 10 g l^−1^; yeast extract 5 g l^−1^; NaCl 5 g l^−1^; bactoagar 13 g l^−1^) containing 100 mg l^−1^ kanamycin, 30 mg l^−1^ rifampicin in the dark for 2 d at 28°C. Two *Agrobacterium* colonies were picked and cultured in 15‐ml falcon tubes filled with 5‐ml YEP liquid medium containing 100 mg l^−1^ kanamycin, 30 mg l^−1^ rifampicin and 200 μM acetosyringone overnight at 28°C. The next day, 2 ml of *Agrobacterium* cells were pelleted in a centrifuge (3000 ***g***) for 4 min and resuspended in the 700A medium (MS basal medium 4.4 g l^−1^; 2,4D 1.5 mg l^−1^; sucrose 68.5 g l^−1^; glucose 36 g l^−1^; acetosyringone 200 μM; pH 5.8). The suspension culture was then transferred to 15‐ml tubes (4–6 ml per tube) and adjusted to 0.35–0.4 at OD_600_ using a spectrophotometer. We gently shook this suspension culture in the dark for > 2–6 h at room temperature (21–25°C) and then immersed all the embryos in 1.8 ml of *Agrobacterium* suspension with 700A infection medium for 5 min. Infected embryos together with *Agrobacterium* suspensions were poured into 562V co‐cultivation plates (N6 basal salt mixture 4.0 g l^−1^; 2,4‐d 2.0 mg l^−1^; sucrose 30 g l^−1^; silver nitrate 1 mg l^−1^; acetosyringone 100 μM; gelrite 2.5 g l^−1^; pH 5.8), and the embryos were spread over the surface by gently shaking the plates by hand. The excess of *Agrobacterium* suspension was then removed from the plates by pipetting and the embryos with scutellum side up were incubated at 20°C in the dark. After 3 d of co‐cultivation, we transferred all embryos (maintaining the scutellum side up) onto the resting medium **605T** (605 medium 11 g l^−1^; casein hydrolyzate 0.3 g l^−1^; 2,4‐D 0.8 mg l^−1^; sucrose 20 g l^−1^; glucose 0.6 g l^−1^; dicamba 1.2 mg l^−1^; silver nitrate 3.4 mg l^−1^; timentin 100 mg l^−1^; cefotaxime 100 mg l^−1^; gelrite 2.5 g l^−1^; pH 5.8) without selection and incubated them at 28°C in the dark. After 7 d, we transferred all embryos to the 605T selection medium with 5 mg l^−1^ bialaphos and incubated them at 28°C in the dark. After an additional 7 d, we transferred embryos with somatic embryos (SEs) on the scutellum side to the shoot formation medium (13329A: MS basal medium 4.4 g l^−1^; zeatin 0.5 mg l^−1^; sucrose 60 g l^−1^; thidiazuron 0.1 mg l^−1^; BAP 1 mg l^−1^; carbenicillin 100 mg l^−1^; bialaphos 5 mg l^−1^ or imazapyr 0.1 mg l^−1^; gelrite 2.5 g l^−1^; pH 5.8). After an additional 1 to 2 wk, we transferred the regenerated shoots with emerging leaves to rooting medium (13158: MS basal medium 4.4 g l^−1^; sucrose 40 g l^−1^; bialaphos 5 mg l^−1^ or imazapyr 0.05 mg l^−1^; gelrite 2.5 g l^−1^; pH 5.8) for 1–2 wk at 28°C in a light chamber (16 h : 8 h, day : night, 20–150 μmol m^−2^ s^−1^; Percival Scientific, Perry, IA, USA) for 2 wk. Rooted plants were acclimated to soil by transferring them to a tray filled with potting media, covering them with a clear plastic dome and maintaining them for 1 to 2 wk in a Conviron GR64 growth chamber (16 h : 8 h, day : night, 20–150 μmol m^−2^ s^−1^) at 28°C. As the plants became more vigorous, we then transplanted individual plants into 5‐gallon (18.9 l) pots containing prewetted soil and maintained them in the glasshouse in standard growing conditions. We verified positive transformed plants by using light applications of Liberty herbicide (0.3%) on 3^rd^–5^th^ leaves (‘BASTA painting’), and scoring resistance (presence or absence of the *Bar* gene) after *c*. 5 d and by construct‐specific PCRs (Table S1).

### Maize leaf regeneration

GGB seeds were surface‐sterilized by placing *c*. 20 mature seeds in a 50‐ml screw‐cap tube and washing them three times with 25 ml of 80% ethanol, vortexing vigorously for 30 s each time. Seeds were then transferred to an autoclaved flask and incubated in 100 ml of 50% Clorox bleach containing 0.1% Tween‐20 for 20 min at 37°C with shaking at 220 rpm, followed by three rinses with 200 ml of sterile water. Sterilized seeds were germinated on MSVS34‐P10 medium (containing 4.4 g l^−1^ MS basal medium, 40 g l^−1^ maltose, 0.1 g l^−1^ casein hydrolyzate, 0.75 g l^−1^ magnesium chloride, 0.5 g l^−1^ glutamine, 1.95 g l^−1^ 2‐ethanesulfonic acid (MES), pH = 5.6, 6 g l^−1^ agar, 3 mg l^−1^ 6‐benzylaminopurine (BAP), 0.1 g l^−1^ ascorbic acid, 10 mg l^−1^ picloram, 100 mg l^−1^ carbenicillin, 10 mg l^−1^ benomyl) for 4 d at 28°C in darkness and were then maintained for an additional 4–6 d at room temperature, also in the dark.

For somatic embryo induction, leaf segments containing the shoot apical meristem region were excised by removing the coleoptile and slicing the tissue into thin sections (0.25–1 mm) with a sterile scalpel. The slices were placed directly onto 605 T medium and incubated at 28°C in darkness for 2 wk, during which SEs developed at wound sites.

For shoot regeneration, tissues containing SEs were transferred onto shoot‐induction medium (13329A) and incubated for 2 wk at 28°C in darkness until shoots developed. Regenerated shoots were moved to rooting medium and grown under a 16 h : 8 h, light : dark photoperiod for 2 wk at 28°C in a growth chamber (20–150 μmol m^−2^ s^−1^; Percival Scientific); media was replaced as needed every 2 wk. Seedlings with well‐developed root systems were transplanted into soil and transferred to a Conviron GR64 growth chamber for continued development.

### Time‐course mRNA‐Seq and analysis

Homozygous GGB‐E1 and WT B104 seeds were germinated as described above, for 7–10 d, then sliced and cultured on 605T medium at 28°C in darkness. Leaf slices were collected at 0, 2, 4, 6, 8 and 10 d, where Day 0 represents tissue collected immediately after cutting. Three biological replicates were collected for each genotype at each time point. Total RNA was extracted using Direct‐Zol RNA Miniprep Kit (ZymoResearch, Tustin, CA, USA), according to the manufacturer's instructions. Libraries were prepared at the CSHL Sequencing Technologies and Analysis Shared Resource using the Mercurius Bulk RNA Barcoding and sequencing (BRB‐Seq) prep kit (Alithea Genomics, Epalinges, Switzerland), and the final pool was sequenced on a NextSeq2000 Sequencer with a Paired‐End 28 × 8 × 8 × 94 run. The raw sequencing data were quality checked using FastQC, and reads were aligned to the maize B73 v.5 reference genome using STAR. Gene‐level read counts were generated from paired‐end BAM files using featureCounts. Count normalization was performed in edgeR using the TMM method. For genotype comparisons at individual time points, differential expression was tested using quasi‐likelihood generalized linear models in edgeR, and genes with false discovery rate (FDR) < 0.05 and |log2FC| ≥ 1 were considered differentially expressed. For time‐course pattern analysis, normalized CPM values were analyzed using maSigPro (Conesa *et al*., 2006). A polynomial regression model of degree 2 was fitted using p.vector with Benjamini–Hochberg correction (*Q* = 0.01), followed by stepwise regression in T.fit (α = 0.05). Significant genes were extracted using get.siggenes with an *R* ^2^ cutoff of 0.5 and grouped into six clusters by hierarchical clustering based on their temporal expression profiles. Gene Ontology (GO) enrichment analysis was performed for the day‐specific differentially expressed genes (DEGs) using public maize GO annotation. Multiple testing correction was performed using the Benjamini–Hochberg method, and GO terms with FDR < 0.05 were considered significantly enriched.

### Asparagine and trehalose treatment assay

To evaluate the effects of asparagine and trehalose on leaf regeneration, leaf slices from homozygous GGB‐E1 seedlings were cultured on four types of 605T‐based media: (1) standard 605T medium containing 20 g l^−1^ sucrose (control); (2) standard 605T medium supplemented with 180 mg l^−1^ L‐asparagine; (3) modified 605T with sucrose reduced from 20 to 5 g l^−1^ and trehalose dihydrate added at 20 g l^−1^; and (4) modified 605T containing 180 mg l^−1^ L‐asparagine, 20 g l^−1^ trehalose dihydrate and 5 g l^−1^ sucrose.

We counted the number of somatic embryos (SEs) produced per seedling after 14 d of culture, when most embryos had developed. SEs were assessed under a dissecting stereomicroscope and scored using morphological criteria. Individual structures were counted as SEs only when they formed discrete, smooth, compact protrusions with clearly defined boundaries relative to the surrounding leaf‐derived tissue or amorphous callus. A pale cream to translucent‐white appearance was used as a supporting feature. For each seedling, leaf tissue was cut into multiple leaf slices, and the SEs produced from all slices were summed and recorded as one value. Each seedling was considered one biological replicate. Treatment effects were analyzed using a factorial negative binomial generalized linear model with trehalose, asparagine, and their interaction as fixed effects. Type III likelihood‐ratio tests were used to assess the significance of the main effects and interaction. Effects with *P* < 0.05 were considered statistically significant.

### mRNA expression

*In situ* hybridization experiments were performed on 2‐wk B104 seedlings fixed in paraformaldehyde (PFA), dehydrated in a graded ethanol series, cleared with Histoclear and embedded in Paraplast. To generate the antisense RNA probes for *PLTP* and *ZmOCL4*, the entire coding sequences together with partial 3′‐untranslated region of each gene were cloned into pENTR223‐Sfi vector. The resulting plasmids were linearized by restriction endonucleases, and the antisense RNA probes (with sizes ranging from 400 to 1000 bp) were synthesized by T7 RNA polymerase (Promega). The vectors, enzymes and primers used for probe design are listed in Table S1.

Quantitative reverse transcription polymerase chain reaction (qRT‐PCR) analysis for *TaGRF4* was performed using an Illumina Eco Real‐Time PCR System. At least three maize seedlings were pooled for each genotype, and three biological replicates were performed for each assay. Total mRNA was extracted using the RNeasy Mini Kit (Qiagen) with on‐column DNase I (Qiagen) treatment and was used for complementary DNA (cDNA) synthesis with a qScript cDNA synthesis kit (Quanta Biosciences, Beverly, MA, USA). qPCR was performed using gene‐specific primers and the PerfeCTa SYBR Green FastMix (Quanta Biosciences). Target cycle threshold values were normalized using ZmUBIQUITIN. Primers used are listed in Table S1.

### Microscopy

For confocal microscopy, the leaf tissues of pZmUBI::mNG‐NLS and pPLTP::BBM*mNG‐NLS transgenic lines as well as ear primordia of pZmOCL4::YFP‐N7 transgenic lines were dissected and placed on slides for direct imaging. Images were obtained by Leica SP8 Confocal Microscope using 514 excitation and 520–575 emission. Images were analyzed using Fiji.

SEM images were obtained from fresh tissue using a JEOL NeoScope JCM‐6000PLUS benchtop SEM.

### Reconstruction of single‐cell atlas of maize seedlings

The cellxgene matrix in the h5ad file format was downloaded from scPlantDB (https://biobigdata.nju.edu.cn/scplantdb/home). We then performed stringent cell‐level quality control and selected highly variable genes before building a latent representation by scVI with default settings (Lopez *et al*., 2018). These highly variable genes were then used for UMAP visualization and Leiden clustering. Clusters with marker genes were identified in an unsupervised manner, and scANVI was applied to refine and harmonize cell‐type annotations.

## Results

### The GGB system is highly efficient for maize embryo transformation

Given that morphogenic factors function in hierarchical order during embryogenesis and meristem formation (Wang *et al*., 2020a; Wu *et al*., 2022), we reasoned that their combination may promote somatic embryogenesis in a synergistic manner, similar to the BBM‐WUS2 system. We therefore tested whether the combination of the maize *BBM* gene (*ZmBBM/EREB53*) and the wheat *GRF4‐GIF1* chimeric gene enhanced maize regeneration during *Agrobacterium*‐mediated embryo transformation, as previously hypothesized (Debernardi *et al*., 2020). We first modified the binary plasmid pBUE411 (pCambia vector backbone; Xing *et al*., 2014) by building a construct containing *ZmBBM* expressed by the *pPLTP* (*PHOSPHOLIPID TRANSFERASE PROTEIN*, Zm00001d023343/Zm00001eb406100) tissue‐specific promoter, which drives expression in embryos and leaves (Takacs *et al*., 2012; Lowe *et al*., 2018; Jones *et al*., 2019), and the wheat *GRF4‐GIF1* chimera driven by the maize *UBIQUITIN1* promoter (Zm00001d015327/GRMZM2G409726; Debernardi *et al*., 2020; Fig. 1a). A CRISPR‐Cas9 cassette and a *BsaI* cloning site for guide RNA (gRNA) cassettes were also included in the original pBUE411 vector for genome editing (pBUE411‐GGB; Fig. 1a). The original gRNA cassette in pBUE411 containing OsU3_promoter‐SmR‐gRNA_scaffold‐OsU3_terminator was replaced by a short DNA fragment carrying double *BsaI* cut sites for single or multiple guide RNA cassette cloning, while the maize codon‐optimized Cas9 gene driven by the *ZmUBI1* promoter was retained from the original vector. We adopted the QuickCorn protocol developed by Corteva (Masters *et al*., 2020), and observed somatic embryogenesis within 1 wk from scutellum cells of immature embryos (Fig. 1b,c) and regenerates plantlets in *c*. 2 months (Fig. 1d,e), comparable to the BBM‐WUS2 system (Lowe *et al*., 2018), and shorter than the 4–5 months usually required in standard transformation protocols (Frame *et al*., 2002).

To test the efficiency of the GGB system, several independent transformations were carried out on immature embryos of the commonly used maize transformable inbred line B104 (Kang *et al*., 2022). Transformation efficiency was calculated as the number of confirmed transgenic plants obtained after treating 100 embryos, roughly corresponding to an independent transformation event. Control transformations were carried out using the same vector backbone (pBUE411) carrying only the *BBM* (pBUE411‐B) gene or the *GRF‐GIF* chimera (pBUE411‐GG; Fig. 1a). Regenerated plantlets were confirmed to be transgenic (carrying the pBUE411 vector with the *bar* resistant gene), first by applying the herbicide Liberty (0.3%) to a section of a mature leaf and scoring the resistance or sensitivity after 5 d (also called BASTA painting technique; Wan *et al*., 1995), and with construct‐specific PCRs. Overall, the efficiency of transformation was *c*. 18% using pBUE411‐GGB in B104. As controls, pBUE411, pBUE411‐GG and pBUE411‐B showed efficiency at 0.9, 1.9 and 2.6%, respectively (Fig. 1f). The efficiency of transformation was thus at least 7‐fold higher than with either component (BBM or GRF‐GIF) in isolation (Fig. 1f; *P* < 0.05), indicating that the synergistic interactions of these morphogenic factors yielded substantial improvement to maize transformation.

We next assessed potential developmental effects of the two morphogenetic factors in the GGB system, and we observed no obvious impact on overall plant growth (Fig. S2). However, fertility tests using reciprocal and self‐crosses revealed skewed pollen transmission in three independent events (designated GGB‐E2 to E4) under both glasshouse and field conditions, with only *c*. 7% of seeds carrying the T‐DNA when transmitted through pollen, compared with *c*. 49.0% when transmitted through ovules (Figs 1g, S2). By contrast, one event (GGB‐E1) had normal pollen transmission (*c*. 47.3% vs 48.9%) when crossed with various genetic backgrounds. This prompted us to examine the integrity of the T‐DNA insertions and the genomic insertion sites in GGB‐E1 to GGB‐E4 plants. Using RNA sequencing of GGB‐E1 and Thermal Asymmetric Interlaced PCR (TAIL‐PCR), we confirmed intact T‐DNA inserts and independent insertion sites for GGB‐E2, GGB‐E3 and GGB‐E4 on chromosomes 8, 10 and 6, respectively. In comparison, GGB‐E1 contained a truncated T‐DNA insertion on chromosome 1 lacking the GIF and NOS terminator sequences, along with a 63 bp deletion at the 3′ end of the coding region (Fig. S3). This suggested that the GRF4‐GIF1 fusion, or that varying levels of *GRF4* expression, impacted fertility by specifically affecting pollen transmission, as reported in rice and tomato (Wang *et al.*, 2024, Mo *et al*., 2023).

### Removal of GIF1 did not reduce transformation efficiency

**Fig. 2:**
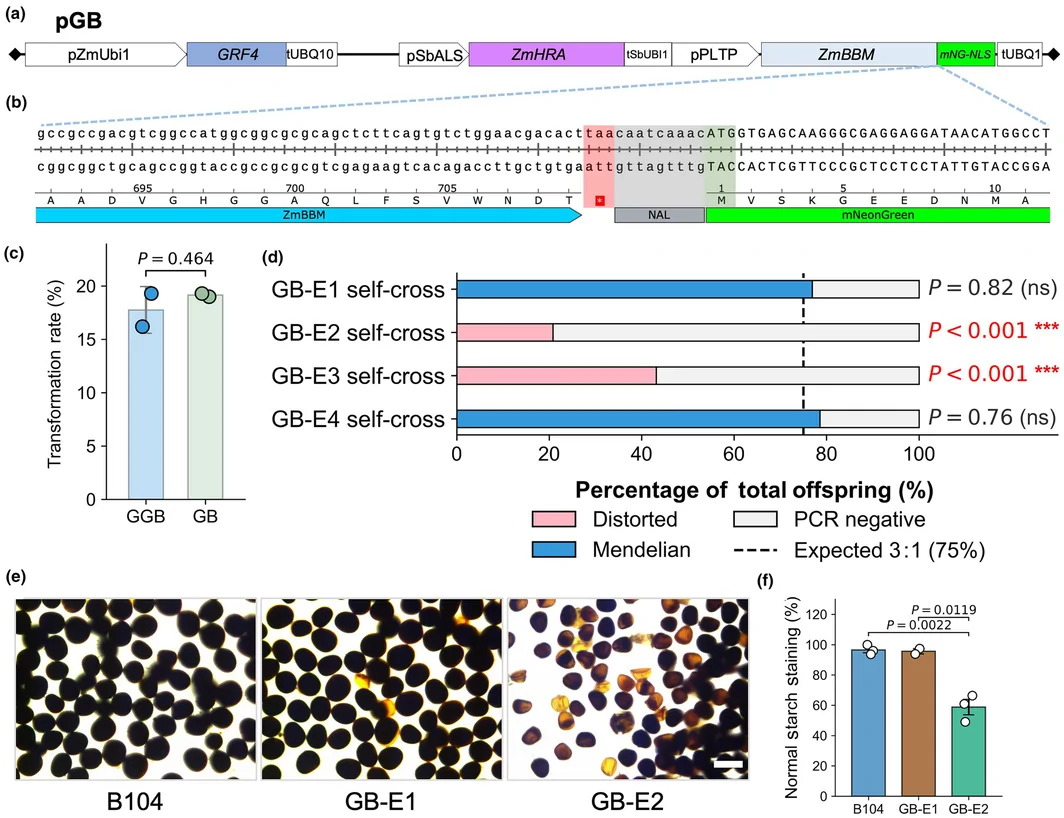
Pollen‐transmission defects are observed in GGB and GB stable insertion lines. (a) Diagram of the GB construct. (b) The mNeoGreen is fused with ZmBBM carrying stop codon by the nucleic acid linker (NAL) CAATCAAAC. (c) Comparison of transformation efficiency between GGB and GB constructs. Transformation efficiency of the pGB transformation delivered by LBA4404 Agrobacterium strain with the helper plasmid pVS1‐VIR2, while pGGB transformation delivered by EHA105 Agrobacterium strain without the helper plasmid pVS1‐VIR2. Two biological repeats for each transformation, fresh embryo no.: 262 for pGB construct, 100 for pGGB construct. Error bars, SD; P, independent two‐sample *t*‐test. (d) Chi‐squared test of GB T‐DNA segregation in the self‐crossed T1 populations. ns indicates nonsignificance. (e) Representative images of pollen starch staining by I_2_‐KI solution in B104, GB‐E1 and GB‐E2 events. Deeply stained (opaque) grains indicate robust starch accumulation, a proxy for pollen maturity and metabolic viability; conversely, light‐brown or translucent grains reflect a lack of starch, indicating developmental failure or reduced viability. (f) Quantification of pollen grains exhibiting normal starch accumulation across B104, GB‐E1 and GB‐E2. B104: pollen number = 551, biological repeat = 3; GB‐E1: pollen number = 707, biological repeat = 2; pollen number = 554, biological repeat = 3. *** indicates statistically significant difference; ns indicates nonsignificance; error bars, SD; P, independent two‐sample *t*‐tests. NAL, nucleic acid linker; NLS, nuclear localization signal; pSbALS, *Sorghum bicolor Acetolactate Synthase* promoter; tUBQ1, *Arabidopsis thaliana UBIQUITIN 1* terminator; tUBQ10, *Arabidopsis thaliana UBIQUITIN 10* terminator. Bar, 100 μm in (e).

To test the hypothesis that the GRF4‐GIF1 fusion was responsible for pollen‐transmission defects, we generated a new ‘GB’ construct by removing the GIF1 sequence and using the pGreen3 backbone (Fig. 2a,b). We also replaced the phosphinothricin acetyltransferase gene Bialaphos selection gene (BlpR), which typically results in a high frequency of escape events (Aesaert *et al*., 2022), with a herbicide‐resistant acetolactate synthase (*HRA*) gene combined for imazapyr selection (Wang *et al*., 2023). In contrast to pBUE411, which is based on a pCambia vector backbone, pGreen3 (pG3) is a nonautonomous vector that requires the ternary helper plasmid pVS1‐VIR2 to supply replication functions and the Vir proteins necessary for T‐DNA transfer into plant cells (Zhang *et al*., 2019). Interestingly, using the pG3‐HRA (pGH3)/ternary pVS1‐VIR2 system, the removal of GIF1 did not impact transformation efficiency (19%) in B104 when compared to the original GGB system (*c*. 19% vs 18%; Fig. 2c). To further assess the efficiency of the GB construct, we tested it in another commonly used transformable inbred line closely related to B73, LH244 (Tan *et al*., 2026). The GB construct achieved a transformation efficiency of 14%, compared with 0.8% for the pG3H empty vector and 3.7% for GGB (Table S2). However, two of the four tested GB events showed similarly skewed T‐DNA segregation (Fig. 2d). We therefore examined pollen starch accumulation via iodine staining in B104, GB‐E1 and GB‐E2 events. The opaque and deeply stained grains indicate robust starch accumulation, whereas light‐brown or translucent grains reflect starch deficiency, associated with compromised viability (Fig. 2e; Datta *et al*., 2002). Quantification of starch‐filled pollen grains showed high frequencies of 96.6% and 95.6% deeply stained pollen in the B104 WT and GB‐E1, respectively. By contrast, the proportion in GB‐E2 was significantly reduced to 58.8% (Fig. 2f). These results suggested that the different level of expression of *GRF4*, rather than the GRF4‐GIF1 fusion itself, may cause distorted T‐DNA segregation.

### Somatic embryogenesis initiates from scutellum cells of immature embryos

**Fig. 3:**
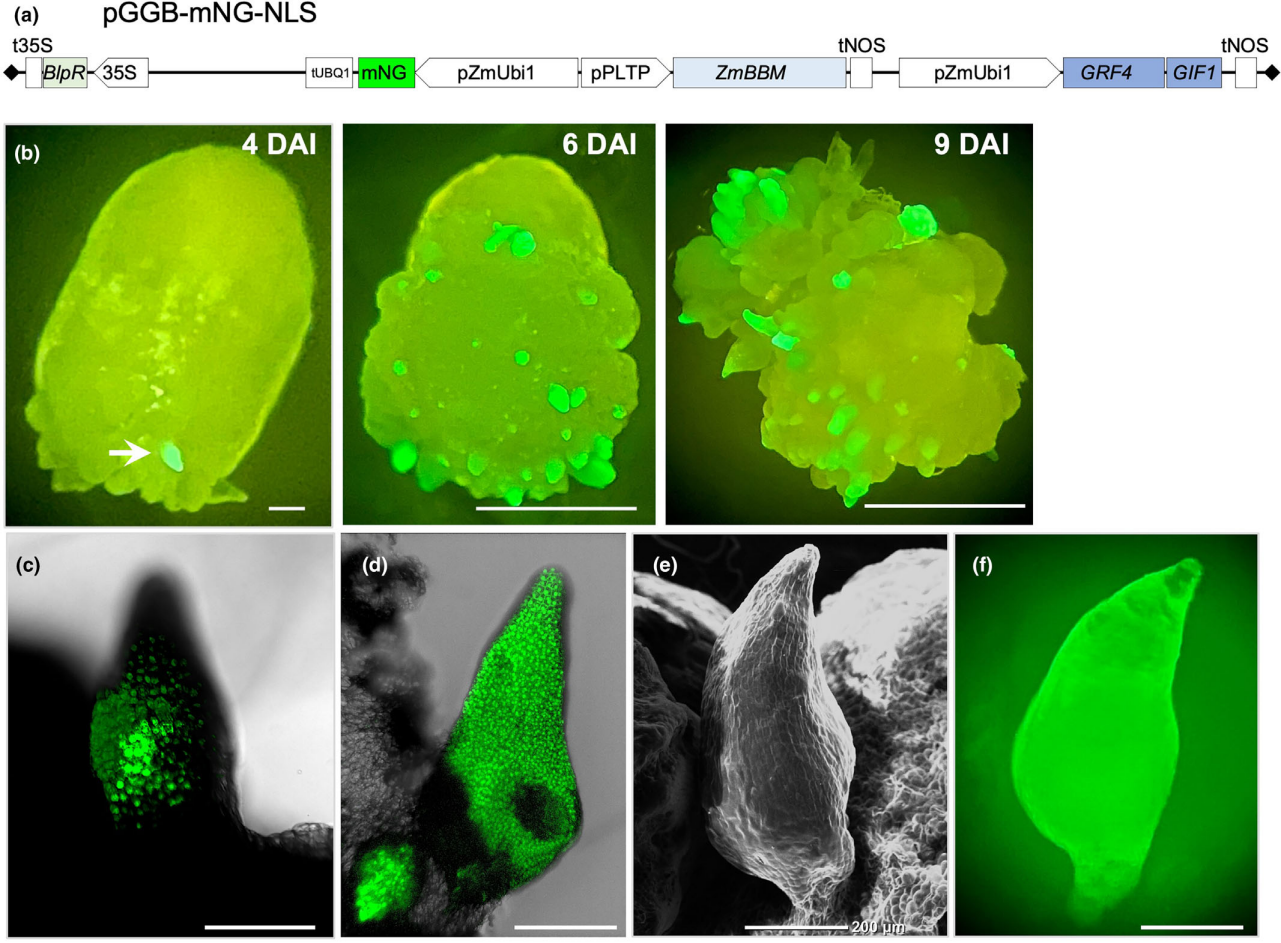
Somatic embryogenesis on the scutella of maize B104 immature embryos. (a) Visualizing somatic embryos on B104 immature embryos using the GGB construct with pZmUBI::mNG‐NLS. mNG, mNeonGreen; NLS, nuclear localization signal peptide. (b) Somatic embryos with green fluorescence signals appeared on the surface of the embryo scutellum at 4, 6 and 9 d after Agrobacterium infection (DAI). The white arrow indicates a somatic embryo expressing mNG proteins. (c, d) Confocal imaging with a Z‐stack of an immature somatic embryo expressing mNG proteins reveals asymmetric mNG signals throughout the embryo at 4 and 9 DAI. (e) Scanning electron microscopy image of an immature somatic embryo at 10 DAI. (f) Stereo fluorescence microscopy image of an immature somatic embryo 10 DAI. Bars, 200 μm in (b) to (f).

To better observe the early stages of somatic embryogenesis, we generated a new construct in which Cas9 was replaced by a nuclear‐localized mNeonGreen (mNG) fluorescent protein driven by the *ZmUBI* promoter (Fig. 3a). This construct was then transformed into immature embryos of B104 following the QuickCorn protocol. As early as 4 d after infection (DAI), we observed short, rod‐shaped structures with green fluorescence signals emerging on the scutella of immature embryos. The number of these structures peaked *c*. 6 DAI and continued to enlarge, becoming more evident by 9 DAI (Fig. 3b). These structures are likely to be somatic embryos, as they show morphogenic features that parallel those of zygotic embryogenesis, progressing from the proembryo stage through the transition stage and into the coleoptilar stage (Fig. S1d–g; Kausch *et al*., 2021).

To examine these structures in detail, we dissected them at 4 DAI and 9 DAI and performed confocal imaging to visualize mNG signals throughout the newly initiated structures, as well as scanning electron microscopy (SEM) to assess their morphology. Confocal imaging using Z‐stacks of the 4 DAI rod‐shaped structures revealed dense mNG foci. By 9 DAI, the mNG signals were more evenly distributed along scutella, indicating somatic embryo development accompanied by enlarged scutella (Fig. 3c,d). SEM and stereo fluorescence microscopy of the rod‐shaped structures at 6 DAI suggested that they resembled embryos at the transition stage (Fig. 3e,f; Wu & Scanlon, 2026). These results confirm that the GGB system enables rapid and reliable somatic embryogenesis from scutellum somatic cells.

### GGB system applications in CRISPR editing and marker line generation

Among the most used applications of transformation are targeted mutagenesis and the spatiotemporal analysis of gene expression in living cells using fluorescent reporters. We first tested CRISPR‐based gene editing of *Zea mays SMALL AUXIN‐UPREGULATED RNA 15* (*ZmSAUR15*; Zm00001d001964/Zm00001eb067430) and its closest paralog, *ZmSAUR79* (Zm00001d026530/Zm00001eb432920), which form a pair of duplicated genes. *SAUR* genes function as primary auxin‐responsive genes and relay hormonal and environmental cues that shape plant growth and development (Ren & Gray, 2015; Vanneste *et al*., 2025). *ZmSAUR15* negatively affects embryonic callus formation in maize, and *ZmSAUR15* loss of function in the recalcitrant inbred line W22 improves its regeneration (Wang *et al*., 2022b).

**Fig. 4:**
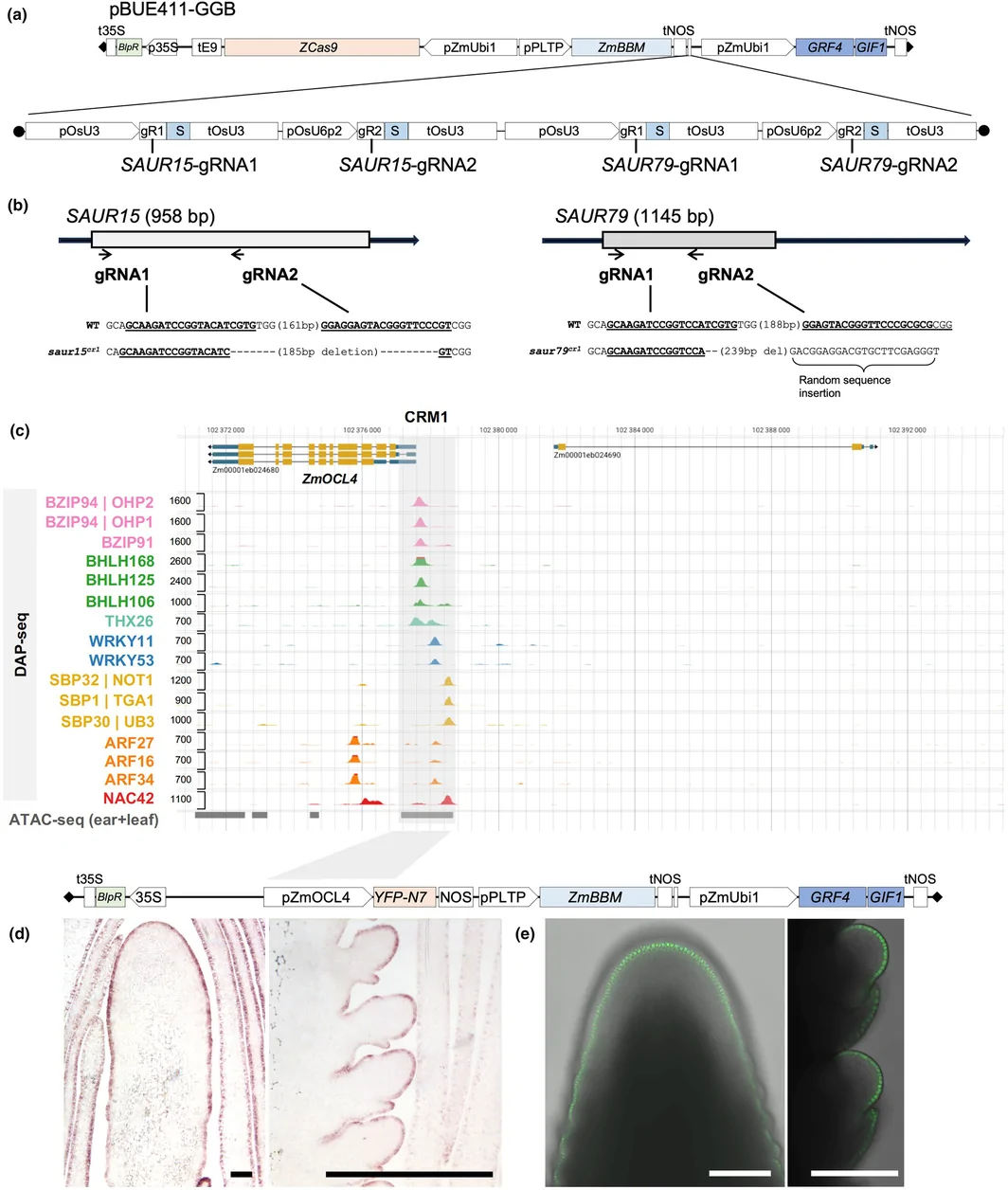
GGB system for genome editing and fluorescence gene expression in maize. (a) Diagram showing the insertion of four guide RNA (gRNA, gR) cassettes into the *Bsa*I cloning site of pBUE411‐GGB construct for CRISPR‐mediated gene knockouts. Two gRNAs targeting *SMALL AUXIN UP‐REGULATED RNA 15* (*ZmSAUR15*) are expressed under the OsU3 and OsU6p2 promoters (pOsU3, pOsU6p2). Similarly, two gRNAs targeting *ZmSAUR79* are also driven by the OsU3 and OsU6p2 promoters. *SAUR15* and *SAUR79* are a pair of duplication genes in the maize genome. *ZmSAUR15 has* previously been shown to impact embryonic callus (EC) formation in maize negatively (Wang *et al*., 2022b). (b) Gene mutations in both *ZmSAUR15 and ZmSAUR79* genes were generated by CRISPR‐Cas9. (c) The *ZmOCL4* promoter, defined by the DAP‐seq CRM, was employed to drive expression of a nuclear‐localized yellow fluorescent protein (YFP). A 1785 bp sequence encompassing the CMR1 region, identified by DAP‐seq peaks from 17 transcription factors within the *ZmOCL4* promoter, was used to recapitulate *ZmOCL4* expression. (d) mRNA *in situ* hybridization of *ZmOCL4* in immature ear primordia revealed that *ZmOCL4* is exclusively expressed in the outermost layer of inflorescence and spikelet meristems, as well as leaves. (e) Confocal imaging of the p*ZmOCL4*::*YFP‐N7* stable transformation line confirmed YFP signals in the outermost layer of inflorescence and spikelet meristems, consistent with the mRNA *in situ* hybridization results. In (a and c), p35S, Cauliflower Mosaic Virus 35S promoter; pPLTP, *Zea mays Phospholipid Transferase Protein* gene promoter; pZmUbi1, *Zea mays UBIQUITIN1* promoter; t35S, Cauliflower Mosaic Virus 35S terminator; tE9, rbcS‐E9 terminator; tNOS, Nopaline Octopine Synthase terminator; tOsU3, *Oryza sativa U3 small nuclear RNA* terminator. Bars, 200 μm in (d) and 100 μm in (e).

For editing of *ZmSAUR15* and *ZmSAUR79* in B104, we designed two guide RNAs (gRNAs) for each gene targeting their single‐exon coding sequences and cloned these four gRNAs into separate gRNA cassettes. We then used Golden Gate assembly to integrate all four gRNA cassettes into the double *Bsa*I site of pBUE411‐GGB (Fig. 4a). The use of two gRNAs per gene enabled us to efficiently generate large deletions in both genes. Sanger sequencing confirmed a 185 bp deletion between the two gRNAs of *ZmSAUR15* and a 239 bp deletion accompanied by a 23 bp insertion in *ZmSAUR79* (Fig. 4b), causing loss‐of‐function mutations in both genes. Double homozygous plants (*Zmsaur15*/*Zmsaur15*;*Zmsaur79*/*Zmsaur79*) were grown under both field and glasshouse conditions, but no obvious defects in plant development were observed.

We next selected *ZmOCL4* (Zm00001d030069/Zm00001eb024680), a gene with a specific expression pattern in the L1 layer in meristems and epidermal cells. *ZmOCL4* encodes an HD‐ZIP IV–type, plant‐specific transcription factor that contributes to both epidermal trichome organization and anther formation in maize (Vernoud *et al*., 2009). Additionally, *ZmOCL4* is often used to define cell types in single‐cell RNA sequencing experiments (Xu *et al*., 2025). To define the promoter sequence of *ZmOCL4*, we first examined the transcription factor binding profile defined by DAP‐seq within a 25 kb region upstream of the coding sequence (Galli *et al*., 2025). This analysis pinpointed a 1785 bp region enriched for binding peaks of multiple transcription factor families, including LBDs, bZIPs, bHLHs, WRKYs, SBPs and ARFs (Fig. 4c). Notably, this region also overlapped with an open‐chromatin region identified by ATAC‐seq (Ricci *et al*., 2019). We cloned this short promoter region and used it to drive nuclear‐localized Yellow Fluorescent Protein (YFP‐N7) by replacing pZmUBI::Cas9 in the pBUE411‐GGB construct (Fig. 4c) and transformed it into the B104 line. Using mRNA *in situ* hybridization, we confirmed the specific expression of *ZmOCL4* in the L1 layer of ear primordia, including the inflorescence meristem, spikelet meristem and young husk leaves (Fig. 4d). Examination of the *pZmOCL4*::*YFP‐N7* transgenic lines revealed L1 specific expression in ear primordia (Fig. 4e). These results confirmed that the promoter region defined by DAP‐seq and ATAC‐seq peaks was sufficient to recapitulate the spatiotemporal specificity of *ZmOCL4* expression.

### Stable GGB insertion lines promote regeneration from leaves

**Fig. 5:**
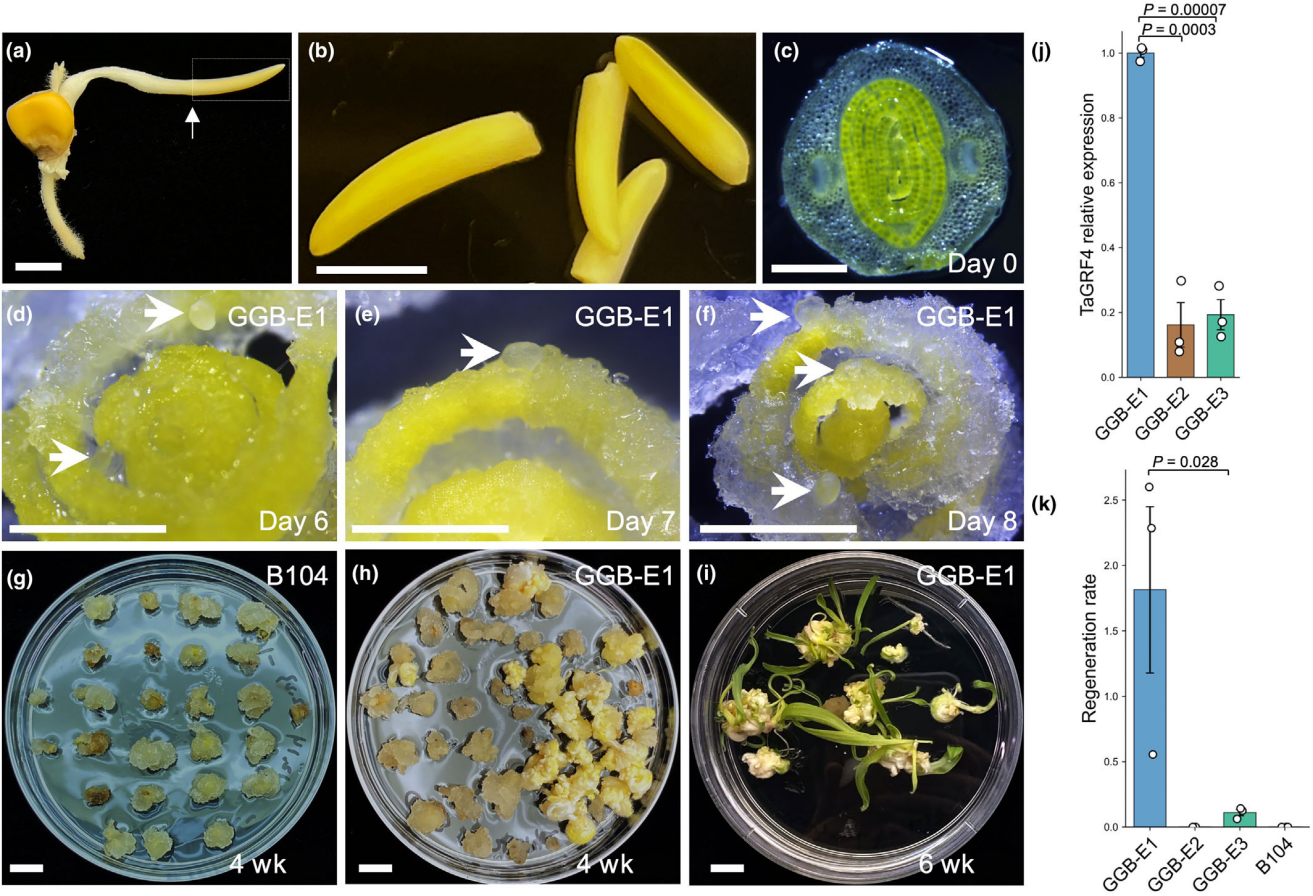
Leaf regeneration from immature leaves of stable maize GGB lines. (a, b) Seedling at the coleoptile stage, 10 d on germination media. Arrow indicate cut site. (c) Hand‐sectioned leaf slices prepared for leaf regeneration. (d–f) Somatic embryos become visible 6–8 d postcultivation on 605T induction media; arrows indicate somatic embryos. (g, h) Vegetative shoots of GGB lines start forming after 1 month of cultivation on 605T and shoot‐induction media, compared with nonembryonic callus propagation in B104 controls. (i) Roots develop from the regenerated shoot on rooting media. (j) Relative *TaGRF4* expression in the leaf base of GGB‐E1, GGB‐E2 and GGB‐E3. Three biological replicates were performed for each genotype; error bars, SD. (k) Comparison of leaf regeneration rates across the GGB‐E1 to GGB‐E3 lines. GGB‐E2 and B104 failed to regenerate from leaves. At least two biological replicates were performed for each genotype; error bars, SD. P, independent two‐sample *t*‐tests. Bars, 1 cm in (a), (b), (g), (h), and (i); 1 mm in (c) to (f).

Immature embryos are widely used as *in vitro* explants for maize transformation because they respond readily to tissue culture, forming embryonic calli that subsequently regenerate shoots (Kausch *et al*., 2021). However, embryos take several months to produce, and their transformation efficiency can be highly variable. By contrast, leaf tissues are easy to produce from germinating seeds, but are notoriously recalcitrant to tissue culture and generally incapable of regeneration in maize (Wang *et al*., 2023). Recent success with leaf transformation using the BBM‐WUS2 system (Wang *et al*., 2023) motivated us to test whether leaves from stably transformed GGB plants could regenerate whole plants. Homozygous GGB‐E1 seeds were germinated on germination medium in the dark for *c*. 10 d until coleoptiles reached 2–3 cm in length (Fig. 5a,b). Coleoptiles with the inner leaves were then dissected and hand‐sliced into thin transverse sections of 0.25–1 mm thickness (Fig. 5c). These slices were placed on 605 T medium, and SEs became visible as early as Day 6 (Fig. 5d–f). B104 leaf slices failed to produce any SEs (Fig. 5g), whereas GGB‐E1 leaf slices generated enlarged SEs on 605T medium (Fig. 5h), which subsequently developed into plantlets following the QuickCorn regeneration protocol (Fig. 5i). We also compared leaf regeneration among GGB‐E1, GGB‐E2 and GGB‐E3 events. GGB‐E1 displayed a regeneration rate of *c*. 181%, calculated as the number of regenerated plantlets relative to the initial number of seedlings. By contrast, GGB‐E2 produced no regenerated plants, and GGB‐E3 showed only modest regeneration at 11%, roughly a 16‐fold reduction compared with GGB‐E1 (Fig. 5k; *P* < 0.05). However, unlike E1 seedlings, the E2 and E3 plants used in these experiments were likely heterozygous, due to pollen‐transmission issues. We also compared expression levels of *TaGRF4* in the leaf base of GGB‐E1, GGB‐E2 and GGB‐E3 heterozygous plants. Heterozygous GGB‐E1 plants had approximately five times higher *TaGRF4* levels than either GGB‐E2 or GGB‐E3 plants (Fig. 5j), suggesting that higher levels of *TaGRF4* expression led to higher efficiency of regeneration from leaf cells.

### Time‐course transcriptome analysis identifies candidate regulators of leaf regeneration

**Fig. 6:**
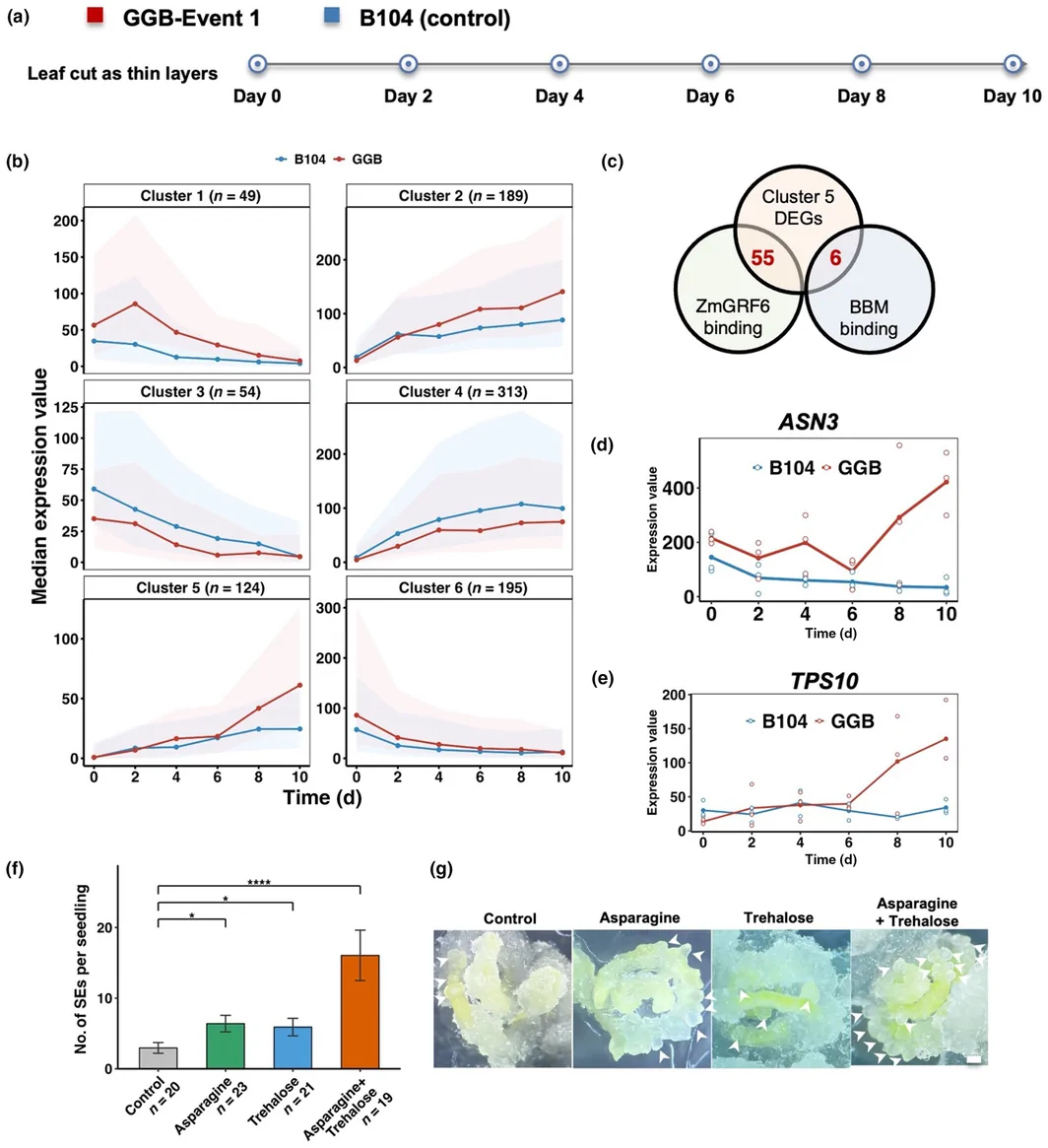
Time‐course transcriptomic analysis of maize leaf regeneration in GGB. (a) Experimental design for time‐course transcriptomic analysis. Sliced shoots of GGB‐E1 and B104 seedlings were cultured on regeneration medium. Tissue was sampled at 0, 2, 4, 6, 8 and 10 d for RNA‐seq analysis. (b) Differentially expressed genes (DEGs) were grouped into six clusters according to their temporal expression patterns. The number of DEGs in each cluster is indicated at the top of each panel. The trend lines indicate the median expression value for each cluster, and shaded areas represent the interquartile range (IQR; 25^th^–75^th^ percentile) across genes within each cluster. (c) Overlap of Cluster 5 DEGs with ZmBBM or ZmGRF6 binding targets identified by DAP‐seq. The red number indicates the gene counts. (d, e) Expression patterns of *asparagine synthetase 3 (ASN3)* and *trehalose phosphate synthase 10* (*TPS10*) during leaf regeneration. (f) Regeneration efficiency, assessed by the number of somatic embryos (SEs) produced per seedling, was enhanced by the addition of asparagine or trehalose to the media and was further increased when both compounds were applied together. Asterisks indicate statistical significance (*, *P* < 0.05; ****, *P* < 0.0001); test performed using a two‐factor negative binomial generalized linear model with asparagine, trehalose and the asparagine‐by‐trehalose interaction as fixed effects. Type III likelihood‐ratio tests were used to evaluate the main effects of asparagine and trehalose and their interaction. Two‐factor analysis indicated significant effects of both compounds, but no significant interaction between the two factors (*P* > 0.05). *n*, number of biological replicates (individual seedlings); error bars, SEM. (g) Representative images of leaf slices cultured on control 605T medium or 605 T media supplemented with asparagine, trehalose or both. Proembryos are indicated by white arrows. Photographs were taken at Days 8–9. Bar, 500 μm.

To investigate the mechanism of leaf regeneration in the GGB system, we performed time‐course mRNA‐seq using homozygous GGB‐E1 regenerating leaves and WT B104 as a control. Leaf tissues were collected at Days 0, 2, 4, 6, 8 and 10 in culture, to identify early molecular markers associated with somatic embryogenesis (Fig. 6a). Principal component analysis showed that the two genotypes shared broadly similar global transcriptional profiles across the time course, and day‐specific differential expression analysis identified genotype‐dependent DEGs (Fig. S4). Notably, genes upregulated in GGB‐E1 at Day 0 were significantly enriched for chromatin and nucleosome assembly related functions (Fig. S4). We next performed trend‐based clustering analysis to identify genes with genotype‐dependent temporal expression patterns during regeneration. This analysis identified 924 DEGs, which were grouped into six distinct clusters across the two genotypes (Fig. 6b). Among these, Cluster 5 was of particular interest because genes in this cluster displayed similar expression patterns in GGB‐E1 and B104 at early stages but became upregulated specifically in GGB‐E1 after Day 6. This temporal pattern suggests that Cluster 5 may contain candidate positive regulators of leaf regeneration as it aligns with the earliest visible proembryo formation (Fig. 5d).

To further identify candidate downstream regulators of GGB leaf regeneration, we compared candidate ZmGRF6 and ZmBBM DAP‐seq target genes with those in Cluster 5 (Fig. 6c; Galli *et al*., 2025). The ZmGRF6 DAP‐seq data, obtained from the maize ortholog of TaGRF4, revealed moderate binding peaks in the regulatory regions of two Cluster 5 genes, *ASPARAGINE SYNTHETASE 3* (*ASN3, Zm00001eb013430*) and *TREHALOSE PHOSPHATE SYNTHASE 10* (*TPS10, Zm00001eb257510*), a noncatalytic member of the TPS family that was also in RNA‐seq Cluster 5 (Fig. 6d,e). Asparagine is a key amino acid for nitrogen metabolism, and regenerable maize callus accumulates higher levels of asparagine than nonregenerable callus (Lozovaya *et al*., 2006). Trehalose‐6‐phosphate functions as a signaling molecule, and noncatalytic TPSs interact with catalytic TPSs and TPPs to regulate plant development (Tran *et al*., 2025). Exogenous application of trehalose also promotes rice regeneration (Gao*et al*., 2025). We therefore tested whether asparagine or trehalose promote leaf regeneration by modifying 605 T medium, which does not contain either compound. Addition of either asparagine or trehalose alone resulted in a modest increase in GGB‐E1 leaf regeneration efficiency (*P* < 0.05; Fig. 6f–g). However, addition of both compounds induced clusters of somatic embryos from leaf slices, and markedly increased regeneration efficiency (*P* < 10^−4^; Fig. 6f–g) from GGB‐E1 leaf slices, but not from WT B104 leaf slices (Fig. S5).

### Leaf regeneration in the GGB system likely initiates from leaf epidermal or sub‐epidermal cells

In both GGB and GB constructs, *TaGRF4* is driven by the maize ubiquitin promoter, whereas *BBM* expression is driven by the tissue‐specific *pPLTP* promoter (Jones *et al*., 2019). To identify the cell types in leaf tissues that express *BBM*, we first performed mRNA *in situ* hybridizations in maize seedlings, including the shoot apical meristem and leaf primordia, using an antisense probe designed against the *PLTP* gene. *PLTP* transcripts were detected predominantly in stomatal cells and in nearby epidermal cells (Fig. S6), while *ZmOCL4* used as a control showed broader expression across the entire leaf epidermis (Fig. S6). We next examined *PLTP* promoter activity in the GB transformation lines, in which mNeoGreen was fused after the ZmBBM stop codon through a nucleic acid linker (NAL) sequence (CAATCAAAC) that permits separate translation of the two genes (Ma *et al*., 2024; Fig. 2b). As a control, a pUBI::mNG‐NLS line showed mNG fluorescence distributed throughout all leaf cell types when imaged by confocal microscopy (Fig. S6). By contrast, the pPLTP::BBM*mNG construct yielded a weak mNG signal in guard cells (Fig. S6).

To further confirm the cell‐type‐specific expression of *PLTP* and *ZmOCL4* genes, we reconstructed published single‐cell atlases of maize shoot apices (Bezrutczyk *et al*., 2021; Satterlee *et al*., 2023). Using the published cellxgene dataset (He *et al*., 2024), we reconstructed the maize seedling atlas from a matrix containing 52 430 cells and 3000 highly variable genes (Fig. S4). UMAP expression plots for *PLTP* and *OCL4*, as well as two stomatal genes *ZmSPCH2a* (Zm00001d016095/Zm00001eb238140), and *ZmMUTE* (Zm00001d009778/Zm00001eb345600) indicated overlapping expression domains within the leaf epidermis (Fig. S6). Retrieving the expression data for *PLTP*, *SPCH2a* and *ZmOCL4* showed that 355 cells co‐express either *PLTP* and *SPCH2a* or all three genes (Fig. S6). Since we cannot exclude that BBM protein can move to nearby cells, these findings suggest that GGB‐mediated leaf regeneration likely occurs in a subset of leaf primordia epidermal or sub‐epidermal cells nearby stomata.

## Discussion

The combination of two morphogenic regulators, BBM and GRF4‐GIF1, in the GGB system provides a simple, rapid and highly efficient approach for maize transformation in the inbred line B104. Although variability in transformation efficiency remains inherent to such experiments, this system nonetheless improves upon the previously reported 6% efficiency achieved in B104 using the QuickCorn protocol with a ternary vector system (Kang *et al*., 2022). Since we observed no major developmental defects aside from reduced pollen transmission, which could be beneficial for transgenic line containment strategies, the GGB system appears well suited for large‐scale applications requiring the rapid generation of multiple transgenic lines, including fluorescent reporter and CRISPR editing lines, as shown in this study. Efficient transformation of the maize B104 inbred was recently reported using the wheat GRF4‐GIF1 or maize GRF1‐GIF1 chimeras in isolation; in both instances, however, the addition of a ternary vector was necessary to achieve high transformation efficiency (Vandeputte *et al*., 2024). Despite this progress, efficient transformation of recalcitrant inbred lines remains a challenge. The GB and GGB systems presented here do not appear to overcome this obstacle, as we were unsuccessful at transforming other inbred lines notoriously difficult to transform (e.g. A619, B73 and W22; Table S2). Efficient transformation was however obtained using two additional lines, the LH244 inbred line and the HiII hybrid (Table S2). LH244 shares *c*. 80% genome‐wide synteny with the B73 reference genome and has *c*. 75% of its protein sequences identical to those of B73 (Tan *et al*., 2026), an historically important genetic background recalcitrant to transformation.

While recent advances in leaf‐based transformation using the BBM/WUS2 system (Wang *et al*., 2023) have expanded the possibilities for regenerating plants from differentiating tissues, the molecular mechanisms governing regeneration from recalcitrant leaf tissues remain largely unresolved. The stable GGB‐mediated transformation system developed here provides robust genetic material for such studies. For example, time‐course RNA‐seq analysis of somatic embryogenesis of our GGB‐E1 line uncovered two additional metabolites, asparagine and trehalose, that together enhance regeneration from leaf tissues. Our fluorescent reporter lines driven by the *ZmUBI1* and *PLTP* promoters, together with *PLTP* mRNA *in situ* hybridization and reanalysis of single‐cell atlases in maize seedlings, indicate that GGB‐mediated leaf regeneration likely initiates within a subset of epidermal or sub‐epidermal cells in developing leaf primordia. In support of this observation, ectopic overexpression of *LEAFY COTYLEDON2* (*LEC2*) in Arabidopsis's cotyledons has been shown to reprogram stomatal‐lineage cells toward a totipotent state by modifying SPEECHLESS‐dependent fate regulation and endogenous auxin biosynthetic pathways (Tang *et al*., 2025), raising the possibility that stomatal‐lineage cells constitute a competent source for leaf regeneration. Additionally, because monocot leaf epidermal cells are competent recipients of Agrobacterium infection and are often the initial sites of viral entry (Xu *et al*., 2023; Chen *et al*., 2025), these properties further position GGB‐targeted epidermal tissues as suitable substrates for both Agrobacterium‐ and virus‐mediated transformation. Collectively, these features offer an expanded toolkit for overcoming long‐standing barriers to transformation in monocot species.

## Competing interests

JMD is co‐inventor in patent US2017/0362601A1 that describes the use of chimeric GRF–GIF proteins with enhanced effects on plant growth (Universidad Nacional de Rosario Consejo Nacional de Investigaciones Científicas y Técnicas). JD and JMD are co‐inventors in UC Davis patent application WO2021007284A2 that describes the use of GRF–GIF chimeras to enhance regeneration efficiency in plants.

## Author contributions

ZC, JZ, DJ and AG designed the research and analyzed data. ZC, JZ, MG, SDI and TC performed the experiments. JMD and JD provided the material for vector construction. ZC, JZ, DJ and AG wrote the paper with contributions from all authors. All authors approved the manuscript. ZC and JZ contributed equally to this work.

## Disclaimer

The New Phytologist Foundation remains neutral with regard to jurisdictional claims in maps and in any institutional affiliations.

## Supporting information


**Fig. S1** Diagrams of two entry vectors to generate individual gRNA cassettes.
**Fig. S2** Morphology of pBUE411 and pBUE411‐GGB plants.
**Fig. S3** Flanking sequences of the T‐DNA insertions in GGB‐E1 to E4.
**Fig. S4** Transcriptomic analysis of leaf regeneration in GGB and B104.
**Fig. S5** Representative images of B104 and GGB‐E1 cultured leaf slices.
**Fig. S6** *PLTP* expression is detected in the shoot epidermis.
**Table S1** Primers used in this study.
**Table S2** Inbred lines and HiII transformation using GGB and GB systems.
Please note: Wiley is not responsible for the content or functionality of any Supporting Information supplied by the authors. Any queries (other than missing material) should be directed to the *New Phytologist* Central Office.

## Data Availability

The pBUE411‐GGB and associated vectors (pBUE411‐GG, pBUE411‐B and gRNA entry vectors) are currently available on Addgene nos.:198233, 198232, 198234, 250279, 250280. The leaf RNA‐seq raw sequencing data have been deposited in the NCBI sequence Read Archive (accession no.: PRJNA1454073).
