# Supplementary material for: The combination of morphogenic regulators BABY BOOM and GRF‐GIF improves maize transformation efficiency and promotes leaf regeneration
Source: New Phytol. 2026 Aug 17;252(1):260–75. doi: 10.1111/nph.71508 (PMC13539940; doi:10.1111/nph.71508)
Supplement: Supplementary file 1 [file NPH-252-260-s001.docx]

**New Phytologist Supporting Information**

**The combination of morphogenic regulators BABY BOOM and GRF-GIF improves maize transformation efficiency and promotes leaf regeneration**

Zongliang Chen, Jiaqi Zhou, Mary Galli, Sessen Daniel Iohannes, Theresa Clark, Juan M. Debernardi, Jorge Dubcovsky, David Jackson and Andrea Gallavotti

**Article acceptance date: 24 July 2026**

**Figure S1.** Diagrams of two entry vectors to generate individual gRNA cassettes.

**Figure S2**. Morphology of pBUE411 and pBUE411-GGB plants.

(**a**) pBUE411 transgenic plant. (**b**) pBUE411-GGB (or GGB) plant. (**c**) Distorted segregation of resistance to Basta herbicide in GGB-E2, GGB-E3 and GGB-E4 self-crossed populations. *** indicate statistically significant difference; P, chi-square test. (**d** and **e**) Somatic embryos are in proembryo stage. (**f**) Transition-stage somatic embryo. (**g**) Coleoptilar-stage somatic embryo.

**Figure S3.** Flanking sequences of the T-DNA insertions in GGB-E1 to E4.

DNA sequences shown in bold blue correspond to the coding sequence of the *GRF4* gene in GGB-E1. DNA sequences shown in bold red correspond to the *GIF1* gene in GGB-E2 to E4. Sequences shown in bold purple represent the tNOS sequence, while the underlined black sequences indicate the genomic flanking regions of the T-DNA insertions. Insertion genome coordinates: GGB-E1, chr6:91607787; GGB-E2, chr8:103922392; GGB-E3, chr10:144848368; GGB-E4, chr6:29954108.

**Figure S4.** (**a**) Transcriptome principal component analysis (PCA) during leaf regeneration in GGB and B104. Each point represents one biological replicate from the time-course RNA-seq dataset, with colors indicating genotype and symbols indicating sampled timepoints. PC1 and PC2 explain 16.9% and 6.6% of the total variance, respectively. (**b-d**) Comparative GO enrichment analysis of time-course DEGs between GGB and B104. Curated Gene Ontology (GO) enrichment patterns for DEGs across the time course, shown for Biological Process (BP), Cellular Component (CC), and Molecular Function (MF), respectively. For each ontology, selected GO terms are displayed across Days 0, 2, 4, 6, 8, and 10. Red indicates stronger enrichment among GGB-upregulated genes, and blue indicates stronger enrichment among GGB-downregulated genes, compared to B104. Color intensity reflects enrichment significance as -log10 (FDR), capped at 10. (**e**) Volcano plots of genotype-dependent differential expression across the regeneration time course. Each point represents a gene. Red and blue points indicate genes significantly upregulated in GGB or B104, respectively, whereas gray points indicate non-significant genes. Labeled genes represent selected annotated DEGs.

**Figure S5.** Representative images of maize B104 (**a** and **b**) and GGB-E1 (**c** and **d**) leaf slices cultured on 605T medium supplemented with trehalose and asparagine at Day 6 (**a** and **c**) and Day 10 (**b** and **d**). White arrowheads indicate discrete, compact structures that were scored as somatic embryos using the morphological criteria described in the Methods. Scale bars, 500 µm. These structures formed in GGB-E1 leaf slices, but not in B104.

**Figure S6.** *PLTP* expression is detected in the shoot epidermis.

(**a** and **c**) Cell-type–specific expression of *PLTP* in the maize shoot is shown by mRNA in situ hybridizations, with strong signals detected in the epidermal cells of leaf primordia and in stomatal cells of young leaves. (**b** and **d**) Cell-type specific expression of *OCL4* in the shoot system is shown by mRNA in situ hybridization, with strong signals detected in the epidermal cells of leaf primordia and young leaves. (**e** and **f**) Compared with pUBI::mNG‑NLS, which displayed mNG fluorescence in pavement cells, subsidiary cells and guard cells, pPLTP::BBM*‑mNG showed fluorescence mostly from guard cells. (**g** - **k**) Single-cell RNA sequencing from leaf tissue and shoot apex tissue showed that *PLTP*, *OCL4*, *SPCH2a* and *MUTE* have overlap expression in leaf lamina epidermis. Bar scales showed 1 mm in (**a** to **d**), 5 µm in (**e**) and 10 µm in (**f**).

**Table S1. Primers used in this study.**

**Table S2. Inbred lines and HiII maize transformation using GGB and GB systems.**
